# The Current Status of the World’s Primates: Mapping Threats to Understand Priorities for Primate Conservation

**DOI:** 10.1007/s10764-021-00242-2

**Published:** 2021-10-31

**Authors:** David Fernández, Daphne Kerhoas, Andrea Dempsey, Josephine Billany, Gráinne McCabe, Elitsa Argirova

**Affiliations:** 1 Department of Applied Sciences, University of the West of England, Bristol, England; 2 Institute of Conservation Science and Learning, Bristol Zoological Society, Bristol, England; 3 West African Primate Conservation Action, Accra, Ghana

**Keywords:** Agriculture, Conservation status, Hunting, IUCN, Logging, Red List

## Abstract

Over the past decades, primate populations have been declining. Four years ago, >60% of species were listed as threatened. As the rate of loss accelerates and new IUCN assessments are being published, we used IUCN Red List assessments and peer-reviewed literature published within the last 5 yr to evaluate the status of primates globally, by region and by taxonomic group. We also examined the main factors affecting a species’ conservation status to determine if we could predict the status of understudied species. We found that 65% of species are in the top three IUCN Red List categories (Vulnerable, Endangered, Critically Endangered). Globally, the main threats to primates are Biological Resource Use, including Hunting & Logging, and Agriculture. The impact of these threats varied by region and taxon. Our model showed that Malagasy and Asian primates, and those affected by Agriculture, Human Disturbance, and Climate Change were more likely to be considered at risk of extinction. The model’s predictive probability, however, was low. Our literature analysis showed that some threats, especially climate change and disease, affected more species than indicated by the IUCN Red List. As we move into the next decade, we must continue tackling hunting and agricultural expansion but also be vigilant about emerging threats. We must also aim to regularly test the effectiveness of mitigation strategies, evaluating their long-term adoption and their impact on primates; as well as to increase communication between researchers and applied conservationists to ensure IUCN assessments include current and emerging threats.

**Supplementary Information:**

The online version contains supplementary material available at 10.1007/s10764-021-00242-2.

## Introduction

Nonhuman primates, our closest biological relatives, make a vital contribution to our planet. Not only do they provide a unique insight into human evolution, biology, behavior, or the emergence and transmission of infectious diseases (Estrada *et al*., 2020), but they also perform key ecological functions, from pollinators and seed dispersers to maintaining community structures across multiple trophic levels (Andresen *et al.*, 2018; McConkey, 2018). However, 4 yr ago, Estrada et al. (2017) found that *ca.* 60% of primate species, representing all 16 families, were threatened with extinction (i.e., classified as Critically Endangered, Endangered, or Vulnerable by the International Union for the Conservation of Nature Red List, hereafter IUCN Red List). Within the four geographical areas where primates are found, i.e., the Americas, Africa, Asia, and Madagascar, 36%, 37%, 73%, and 87% of primate species were threatened, respectively, although their conservation status within each country varied greatly (Estrada *et al*., 2017). In China alone, for instance, 80% of its primate were threatened, with >15 of those species having fewer than 3000 individuals remaining, and 2 gibbon species (*Hylobates lar* and *Nomascus leucogenys*) having been extirpated from this region in the past decade (Li *et al*., 2018). The work of Estrada *et al*. (2017) was completed recently, and over the past decade there has been some important primate conservation successes, such as the downgrading of the mountain gorilla (*Gorilla beringei* ssp. *Beringei*) from Critically Endangered to Endangered (Hickey *et al.*, 2020). However, threats that primates face are severe and can rapidly have strong impacts on populations. In addition, Estrada’s study (2017) was largely based on IUCN Red List data published in 2008. Thus, further studies are needed to examine the current status of primate conservation in light of the recently published IUCN Red List assessments for the majority of primate species, as well as the rapidly changing landscapes that directly impact threatened species around the world.

Human population growth and associated increases in human activities, such as urban expansion, hunting, trade, logging, climate change, diseases transmission, fossil fuel extraction, mining, infrastructure development, tourism, and threats to human–primate coexistence (including persecution killing)—most of which are examined in detail in this special issue—jeopardize primate existence, as many countries struggle to balance economic development with biodiversity protection (Boonratana, 2020; Estrada *et al*., 2017, 2020; Li *et al.*, 2018). In particular, the loss of tropical forests, home to most primate species, is one of the main factors contributing to their decline (Estrada *et al.*, 2017, Hanski *et al.*, 2013). However, drivers of forest loss vary regionally. For example, between 2001 and 2015, commodity-driven deforestation (e.g., soybean, palm oil, rubber, mining, etc.), which involves long-term forest conversion, was the leading cause of forest loss in Asia (78%) and the Americas (56%), while it was responsible for only 4% of forest lost in sub-Saharan Africa (Curtis *et al*., 2018). Instead, the main driver of forest loss in the latter region was small-size agriculture (92%), characterized by short-term forest clearing followed by forest regrowth, which represented only 9% and 31% of the deforestation in Southeast Asia and the Americas, respectively (Curtis *et al*., 2018).

As human activities and threats vary widely in different regions of the world, so do primate species susceptibility to such threats (Isaac & Cowlishaw, 2004). Such vulnerability can depend on several species-specific traits that are associated with different specialisms (McKinney, 1997), such as population size, diet, and ecological flexibility (Boyle & Smith, 2010; Isaac & Cowlishaw, 2004; Purvis *et al*., 2000). For example, species with small populations, such as the Roloway monkey (*Cercopithecus roloway*), a Critically Endangered Afro-Eurasian monkey with fewer than 2000 individuals remaining (Koné *et al*., 2019), are more susceptible to demographic stochasticity and local catastrophes and have a higher risk of extinction than those with larger population sizes (Purvis *et al*., 2000). Diet specialists are also considered at risk (Jervall & Wright, 1998), such as the Critically Endangered greater bamboo lemur (*Prolemur simus*) in Madagascar, with a diet that consists almost exclusively of bamboo (Tan, 1999), compared to the ring-tailed lemur (*Lemur catta*), an opportunistic omnivore that exhibits greater dietary flexibility, changing its primary food resource in response to ecological unpredictability (Gould & Sauther, 2007). Thus, intrinsic risk factors must be considered when determining risk of extinction.

The goals of this article therefore are to 1) report on the current conservation status of the world’s primates; 2) analyze the main threats to primates as reported in the IUCN Red List by region (the Americas, Africa, Asia, Madagascar) and by taxonomic group (family, subfamily, tribe); and 3) model the most important factors influencing a species’ IUCN conservation status to help inform the development of conservation strategies for poorly known species. Finally, because the current primate IUCN Red List is based on assessments that took place between 2013 and 2019, as well as some from as far back as 2008, we also 4) compare threats identified in the current IUCN Red List assessments with those appearing in recent (i.e., 2015–2020) scientific publications, to identify emerging threats that may need further attention.

## Methods

### Data Collection

#### IUCN Red List Assessment

On July 9, 2020, we extracted information on all primate species recorded on the IUCN Red List website (IUCN, 2020). For some species from Africa, Asia, and Madagascar, July 9 was the day the most recent Red List assessment was published on the IUCN website. Unfortunately, the updated Neotropical primate assessments had not been published at the time this manuscript was submitted. Although the Red List evaluates primates at the subspecies level, in this study we used species as the level of analysis. In total, we included 491 species.

For each primate species, we extracted the common and scientific name; the assessment publication year; the IUCN threat category (hereafter called conservation status, e.g., Critically Endangered); the region (i.e., The Americas, Africa, Asia, or Madagascar); the countries of the species’ extant geographical range excluding “extant introduced,” “possibly extinct,” and “extinct” and the IUCN Level 1 and Level 2 conservation threats (https://www.iucnredlist.org/resources/threat-classification-scheme; Tables I and II). Unfortunately, for most primate species there was no information on the threats’ timing, scope, and severity, but merely the type of threats faced by each species.

**Table I:** Number of species within each region, primate group and overall affected by each of the IUCN Red List major conservation (Level 1) threat category

				Level 1 IUCN threat category											
			Number of species	Residential & Commercial Development	Agriculture & Aquaculture	Energy Production	Transportation & Service Corridors	Biological Resource Use	Human Intrusion & Disturbance	Natural System Modifications	Invasive Species, Genes & Diseases	Pollution	Geological events	Climate Change & Severe Weather	Other
*Region*															
	Africa		106	26	89	38	27	91	25	11	7	4	0	19	1
	Asia		116	56	106	22	26	112	11	38	7	7	2	3	0
	Madagascar		107	5	104	31	1	86	0	37	1	0	0	7	0
	The Americas		162	68	93	16	38	110	8	19	6	1	0	3	0
		*Average*		38.8	98.0	26.8	23.0	99.8	11.0	26.3	5.3	3.0	0.5	8.0	0.3
		*SD*		24.8	7.2	8.4	13.5	11.4	9.0	11.6	2.5	2.7	0.9	6.6	0.4
*Primate group*															
	Cheirogaleidae		40	0	38	10	0	26	0	8	0	0	0	0	0
	Daubentoniidae		1	0	1	0	0	1	0	1	0	0	0	0	0
	Indriidae		19	0	18	7	1	16	0	7	1	0	0	2	0
	Lemuridae		21	0	21	10	0	21	0	12	0	0	0	4	0
	Lepilemuridae		26	5	26	4	0	22	0	9	0	0	0	1	0
	Galagidae		19	4	12	2	1	10	0	2	1	0	0	1	0
	Lorisinae		11	5	11	0	3	10	1	6	1	1	0	0	0
	Perodicticinae		5	1	4	1	0	5	0	1	0	0	0	0	0
	Tarsiidae		10	3	10	1	0	9	1	2	4	5	2	0	0
	Alouattinae		14	11	12	2	4	14	0	1	1	0	0	2	0
	Atelinae		11	8	10	4	3	11	2	4	0	0	0	0	0
	Aotinae		11	9	9	3	4	9	2	1	0	0	0	0	0
	Callitrichinae		49	18	30	4	12	30	1	6	4	0	0	1	0
	Cebinae		17	7	7	0	2	11	1	1	1	0	0	0	0
	Saimiriinae		5	1	4	0	2	4	0	1	0	0	0	0	0
	Callicebinae		31	11	16	2	9	9	2	3	0	1	0	0	0
	Pitheciinae		24	3	5	1	2	22	0	2	0	0	0	0	0
	Cercopithecini		35	11	30	12	9	32	6	1	1	3	0	6	0
	Papionini		41	9	32	11	8	38	7	4	2	1	0	6	0
	Colobinae		74	34	69	21	23	72	17	24	1	1	0	2	1
	Hylobatidae		20	9	20	5	4	20	2	5	0	0	0	0	0
	Ponginae		3	3	3	3	2	3	0	3	0	0	0	3	0
	Homininae		4	3	4	4	3	4	2	1	4	0	0	4	0
		*Average*		6.7	17.0	4.7	4.0	17.3	1.9	4.6	0.9	0.5	0.1	1.4	0.0
		*SD*		7.4	15.1	5.0	5.2	15.2	3.7	5.1	1.3	1.2	0.4	1.9	0.2
*Overall*															
	Total		491	155	392	107	92	399	44	105	21	12	2	32	1
	%			31.6	79.8	21.8	18.7	81.3	9.0	21.4	4.3	2.4	0.4	6.5	0.2

Biological Resource Use and Agriculture and Aquaculture were the most predominant, followed by Residential & Commercial Development, Energy Production, Natural System Modifications, and Transportation and Service Corridors. Primate groups included family, subfamily, and in the case of the subfamily Cercopithecinae, tribe.

**Table II:** Number of species within each region and primate group and overall affected by specific IUCN Level 2 threats types within Agriculture & Aquaculture and Biological Resource Use, the two IUCN Level 1 threat categories most predominant among primates

				Agriculture & aquaculture Level 2 threats				Biological resource use Level 2 threats			
			Number of species	Annual & Perennial Non-timber Crops	Wood & Pulp Plantations	Livestock Farming & Ranching	Marine & Freshwater Aquaculture	Hunting & Collecting Terrestrial Animals	Gathering Terrestrial Plants	Logging & Wood Harvesting	Fishing & Harvesting Aquatic Resources
*Region*											
	Asia		116	98	39	7	0	103	3	88	3
	Africa		106	86	18	32	1	79	3	74	0
	Madagascar		107	96	3	18	0	70	4	60	0
	The Americas		162	88	11	78	0	75	2	100	0
		*Average*		92.0	17.8	33.8	0.3	81.8	3.0	80.5	0.8
		*SD*		5.1	13.4	27.0	0.4	12.7	0.7	15.0	1.3
*Primate group*											
	Cheirogaleidae		40	38	0	1	0	14	1	18	0
	Daubentoniidae		1	1	0	0	0	1	0	1	0
	Indriidae		19	15	2	5	0	15	0	11	0
	Lemuridae		21	19	1	5	0	20	2	18	0
	Lepilemuridae		26	23	0	7	0	20	1	12	0
	Galagidae		19	9	3	4	0	1	0	10	0
	Lorisinae		11	7	6	1	0	10	0	6	1
	Perodicticinae		5	4	1	1	0	4	0	3	0
	Tarsiidae		10	10	1	0	0	7	0	8	0
	Alouattinae		14	10	0	11	0	14	0	12	0
	Atelinae		11	10	2	9	0	11	0	10	0
	Aotinae		11	9	0	7	0	5	1	8	0
	Callitrichinae		49	28	4	26	0	6	1	30	0
	Cebinae		17	7	3	6	0	11	0	11	0
	Saimiriinae		5	4	1	3	0	1	0	4	0
	Callicebinae		31	16	1	12	0	5	0	7	0
	Pitheciinae		24	4	0	4	0	22	0	18	0
	Cercopithecini		35	30	7	11	0	32	1	23	0
	Papionini		41	31	7	6	1	37	1	27	1
	Colobinae		74	66	21	12	0	65	2	61	0
	Hylobatidae		20	20	10	2	0	19	2	17	1
	Ponginae		3	3	1	0	0	3	0	3	0
	Homininae		4	4	0	2	0	4	0	4	0
		*Average*		16.0	3.1	5.9	0.0	14.2	0.5	14.0	0.1
		*SD*		14.7	4.7	5.7	0.2	14.4	0.7	12.5	0.3
*Overall*											
	Total		491	368	71	135	1	327	12	322	3
	%			74.9	14.5	27.5	0.2	66.6	2.4	65.6	0.6

Primate groups included family, subfamily, and in the case of the subfamily Cercopithecinae, tribe.

#### Scientific Publications

We also investigated current threats faced by primate species through a search of peer-reviewed scientific articles published between January 1, 2015 and July 1, 2020 to examine if there were new and emerging threats that had become more prevalent after the last IUCN Red List assessment. We based these threat categories on a preliminary literature review of previous publications retrieved from a Google Scholar search for “major drivers of primate decline” and other primate threat terms (e.g., hunting). In total, we identified 95 specific keywords from the literature review, which we organized into 12 predefined, higher-level categories: Urbanisation & Road Development; Commercial Agriculture; Small-Holder Agriculture; Energy Production & Mining; Logging, Wood Harvesting, & Gathering of Terrestrial Plants; Commercial Hunting; Subsistence Hunting; Pet Trade; Civil Unrest; Genes; Diseases; and Climate Change & Severe Weather (Electronic Supplementary Material [ESM] Table SI).

We then conducted a second Google Scholar search for each of the 12 predefined, higher-level categories using all the keywords associated with that category separated by “OR,” followed by “primate conservation” AND “threat.” We used speech marks when keywords associated with threat categories were phrases rather than one word. For example, for the threat category “Urbanisation & Road Development” the search terms were Road OR Rail OR “Human population” OR “Illegal settlement” OR “Expansion of urban area” OR “Infrastructure development” OR Encroachment OR Urbanisation OR Urbanization AND “Primate conservation” AND “threat.” Some threat categories contained too many threats to fit in a single Google Scholar search and we therefore split into separate searches (ESM Table SII).

We collected a total of 8977 articles across the 12 predefined, threat categories. Given that the data we needed from this primary search required manual extraction, it was not feasible to sample the total search. Therefore, after each Google Scholar search, we sampled only 10% of all articles retrieved for each search term (ESM Table SII), selecting only primary research articles (namely peer-reviewed papers, PhD and master’s theses) that discussed conservation threat(s) to primates (*N* = 899 articles). We deemed the 10% cutoff appropriate, as beyond 10% most articles were not relevant to the present study, despite containing the search terms. Out of the 899 articles that came up in the combined search results, many (55.7%) articles had been selected more than once. After excluding duplicates, the total number of articles reviewed was 398. For each article, we read its title, abstract, and keywords, and when needed the whole article and extracted the following information: article title, first author’s name, year of publication, scientific and common name of the species and subspecies studied, country, region, and the type(s) of aforementioned threats affecting each primate species, including those threats based on anecdotal evidence or those cited in the publication but for which no reference was provided. We combined entries for subspecies with their respective species’ entry. In total, we extracted information for 287 species from the peer-reviewed literature.

We then grouped all extracted threat types into 12 larger threat categories. During the second Google Scholar search it became apparent that the predefined, higher-level threat categories did not accurately capture the threats to primates being discussed in the literature. Thus, we modified these higher-level threat categories to better suit what was described in the literature. These new 12 threat categories were Urbanisation & Road Development, Commercial & Small-Holder Agriculture; Habitat Degradation; Energy Production & Mining; Logging, Wood Harvesting, & Gathering of Terrestrial Plants; Hunting; Tourism; Pet Trade; Civil Unrest; Genes; Diseases; and Climate Change & Severe Weather (see ESM Table SIII for a full list of terms). Many terms found in the papers were ambiguous and/or very broad (e.g., resource extraction, anthropogenic activities). In most cases, we assigned those terms under the Habitat Degradation category.

### Analysis

#### IUCN Red List Assessment

To investigate the main threats affecting primates, we examined the distribution of the IUCN conservation status and of major conservation threats (Level 1) across regions and across primate taxonomic groups. We also examined the distribution of specific type of threats (Level 2) for those Level 1 threats that were most prevalent. Taxonomic groups included family, subfamily, and in the case of the subfamily Cercopithecinae, tribe (i.e., Cercopithecini, Papionini), as in many cases using the family taxonomic grouping would merge subfamilies with markedly different biology and ecology (e.g., subfamilies of Cercopithecinae and Colobinae). In the case of Cercopithecinae, we used tribes to capture differences in the ecology, biology, and distribution that exists between Cercopithecini (i.e., typically smaller-bodied primates restricted to Africa) and Papionini (i.e., mainly mid- [5–10 kg] to large-bodied [≥10 kg] species living in Africa and Asia).

#### Modeling IUCN Red List Status

To examine which factors were most predictive in determining a species’ IUCN Red List conservation status, we performed an ordinal logistic regression (Agresti, 2002). The response variable was conservation status, with five levels (Least Concern, Near Threatened, Vulnerable, Endangered, Critically Endangered). Predictor variables included the region of occurrence (the Americas, Africa, Asia, Madagascar), whether the species was reported to be affected by any of the 12 IUCN Level 1 threat types (Yes/No), the number of countries in which the species was found, and the number of Level 1 threats described for this species. We excluded from the analysis species classified as Data Deficient and those for which there was no IUCN threat type described. In total, we included 438 species.

The model initially included all predictor variables. To improve the predictive power of the model, we removed variables with the lowest explanatory power using a backward stepwise procedure (Miller & Forte, 2017). We maintained the model with the lowest Akaike information criteria. To test for the accuracy of our model, we repeated model selection using a subset of our original dataset, which included a random selection of 80% of the species. We tested the proportion odds assumption on the resulting model by running binary logistic regressions with varying cross points on the response variable (UCLA: Statistical Consulting Group, 2020) which indicated that the assumption might not be met for all variables. Results of the model therefore should be interpreted with caution.

#### Scientific Publications

We used a similar process for the scientific publications analysis as described for the analysis of IUCN threat categories.

We run all analyses in R for Windows (version 4.0.2, R Core Team, 2020) using the packages cat (Schafer, 2012) and MASS (Venables & Ripley, 2002).

## Ethical Note

The authors declare that they have no conflict of interest.

### Data Availability

The datasets during and/or analysed during the current study available from the corresponding author on reasonable request.

## Results

### IUCN Red List Assessment: Distribution of IUCN Conservation Status

Out of the 491 extant species of primates included in the IUCN Red List as of July 9, 2020, 319 (65.5%) were threatened with extinction, meaning they fall within the top three categories of the IUCN Red List (Vulnerable, Endangered, Critically Endangered). In particular, 100 species (20.4%) were Vulnerable, 136 (27.7%) were Endangered, and 83 (16.9%) were classified as Critically Endangered. In contrast, 118 species (24.0%) were classified as Least Concern and 32 (6.5%) as Near Threatened. There was not enough information to assess the status of 22 species (4.5%), which were classified as Data Deficient.

**Fig. 1:**
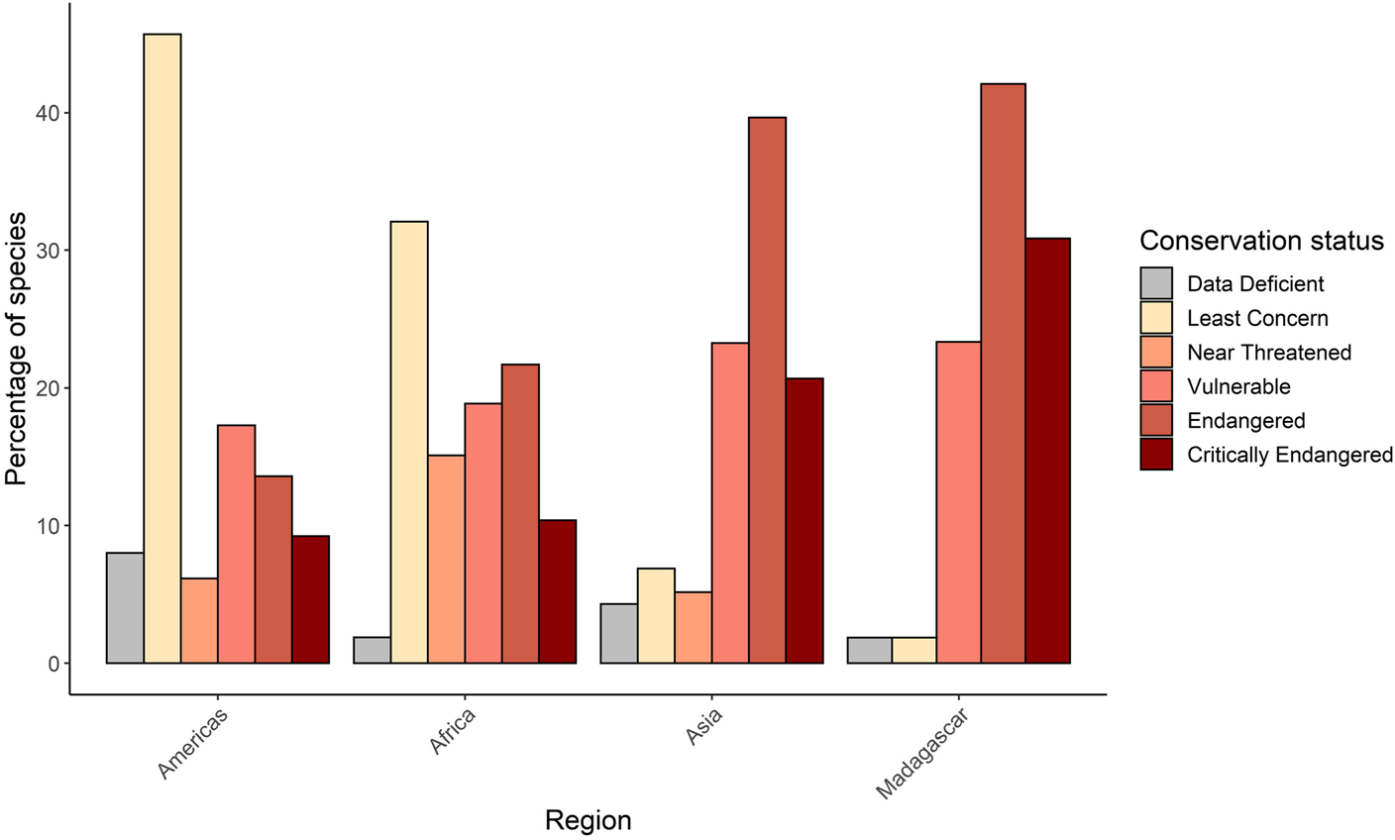
Proportion of primates within each of IUCN’s conservation status across the four regions where primates are found. Data included the 491 species included in the IUCN Red List as of July 9, 2020. A species is considered Threatened when it is classified as Vulnerable, Endangered, or Critically Endangered.

**Fig. 2:**
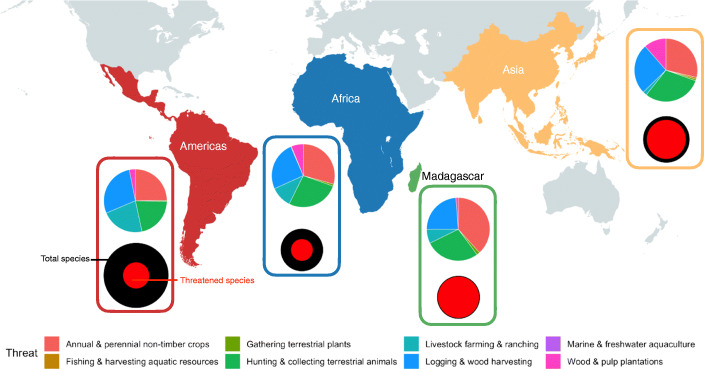
Main threats and conservation status within each of the four primate regions based on IUCN data. *Top circle*: proportion of species affected by specific Level 2 threat types for the two most predominant threats affecting primates globally, i.e., “Biological Resource Use” and “Agriculture & Aquaculture.” *Bottom circle*: proportion of threatened species, i.e., classified as Vulnerable, Endangered, or Critically Endangered.

Across regions, Madagascar (*N* = 103 species, or 96.3%) and Asia (*N* = 97 species, or 83.7%) had the largest proportion of threatened species (Figs. 1 and 2). These were followed by Africa and the Americas, with 54 (50.9%) and 65 (40.2%) threatened species, respectively (Figs. 1 and 2). In the Neotropics, most primates (i.e., 51.9%, *N* = 84 species) were classified as either Least Concern or Near Threatened (Figs. 1 and 2).

**Fig. 3:**
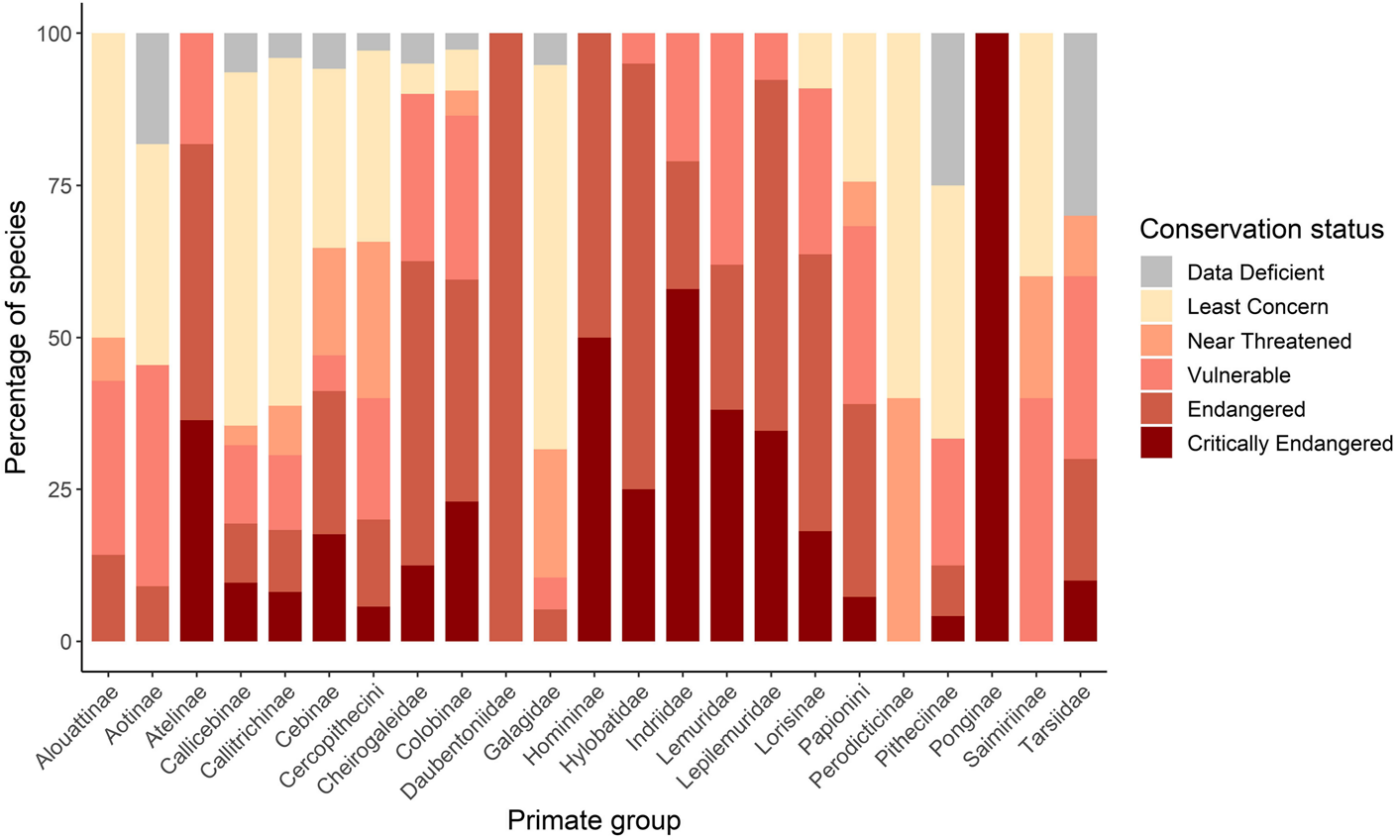
Proportion of species within each IUCN Conservation Status for each primate taxonomic group (i.e., family, subfamily, and in the case of the subfamily Cercopithecinae, tribe) included in the study. Primate groups are presented in alphabetical order.

Among taxa results also varied, with the percentage of species classed as threatened ranging from 0% to 100% (mean = 65.0 ± SD 31.8). More specifically, all species within Atelinae (*N* = 11 species), Indriinae (*N* = 19 species), Daubentoniidae (*N* = 1 species), Homininae (*N* = 4 species), Hylobatidae (*N* = 20 species), Lemuridae (*N* = 21 species), and Lepilemuridae (*N* = 26 species) were considered threatened. In contrast, for Callicebinae (*N* = 30 species), Pitheciinae (*N* = 24 species), Callitrichinae (*N* = 49 species), and Galagidae (*N* = 19 species) only between 10.5% and 33.3% of their species were considered threatened (Fig. 3). None of the five species of Perodicticinae were threatened (Fig. 3).

### IUCN Red List Assessment: Distribution of IUCN Threat Types

Out of the 12 Level 1 threats used by the IUCN, the most predominant were Biological Resource Use and Agriculture, affecting a total of 399 (81.3%) and 392 (79.8%) species, respectively (Table I). These were followed by Residential & Commercial Development (*N* = 155 species, or 31.6%), Energy Production (*N* = 107 species, or 21.8%), Natural System Modifications (*N* = 105 species, or 21.4%), and Transportation and Service Corridors (*N* = 92 species, or 18.7%). When we examined the specific Level 2 activities within these two threats, we found that the most prevalent Biological Resource Use activity was Hunting (*N* = 327 species, or 81.8%), followed closely by Logging (*N* = 322 species, or 80.5%). The most prevalent Agricultural Level 2 activity was Non-Timber Crop Production, affecting on average 368 (92.0%) of the species, followed by Livestock Production, which affected 135 species (33.8%) (Table II).

At the regional level, we found that all four regions were affected by at least 8 different Level 1 threats (Table I): Madagascar was affected by 8, the Americas by 10, and Africa and Asia by 11 each. Although the most common threats to primates globally were Biological Resource Use and Agriculture, there was variation among regions. For example, in the Neotropics, Biological Resource Use (*N* = 110 species, or 67.9%) and Agriculture (*N* = 93 species, or 57.4%) were the most prevalent threats. The number of species impacted by these threats in the Americas, however, was lower compared to the other three regions. Similarly, in Madagascar, the impact of Development, Transport, and Human Intrusion was much lower than in other regions, affecting only 5 (4.7%), 1 (0.1%), and 0 species, respectively. Finally, in Africa Human Intrusion (*N* = 25 species, or 23.6%) and Climate Change (*N* = 19 species, or 17.2%) affected a relatively large proportion of species compared to a mean of 4.8 ± SD 3.9% (range: 0.0–9.5) and 3.7 ± SD 2.1 (range: 1.9%–6.5%) of species in the other three regions (Table I).

The most predominant activity within the Agriculture Level 1 threat category was the Annual and Perennial Non-Timber Crop Production, affecting a total of 386 species (74.9%). This threat was particularly prevalent in Africa, Asia, and Madagascar, where it affected 280 (85.1%) species (Table II, Fig. 2). Non-timber crop production was a much less prevalent threat for Neotropical primates, however, affecting a relatively lower number of species (*N* = 88 species, or 54.3%; Table II, Fig. 2). In contrast, Livestock Farming & Ranching was much more prevalent in the Americas than in other regions, affecting 75 species (48.2%) of primates, compared to Asia, Africa, and Madagascar, where this activity affected only 57 (17.3%) species across these three regions combined (Table II, Fig. 2).

Within the Biological Resource Use Level 1 threat category, in Africa, Asia, and Madagascar, Hunting was the main activity, affecting 252 (76.6%) of the species, while it was less common in the Americas, where hunting affected 75 (46.3%) species (Table II, Fig. 2). The prevalence of Logging and Wood Harvesting, however, was comparable across all regions, with a mean of 80.5 ± SD 15.0 species (range: 60–100 species, or 56.1%–75.9%) being affected (Table II, Fig. 2).

When examining the distribution of Level 1 IUCN threats across primate taxonomic groups, we found that at least 3 different threat types affected all 23 groups: 7 were affected by 3–6 threat types (Daubentoniidae, Cheirogaleidae, Lemuridae, Perodicticinae, Saimiriinae, Lepilemuridae, and Pitheciinae), 13 by 7–9 threat types (Aotinae, Atelinae, Cebinae, Hylobatidae, Indriidae, Ponginae, Alouattinae, Callicebinae, Galagidae, Lorisinae, Callitrichinae, Homininae, and Tarsiidae) and 3 by 10 or more (Cercopithecini, Papionini and Colobinae) (Table I).

We found that the most common threats across primate taxonomic groups were not always Biological Resource Use and Agriculture (Table I). For example, Agriculture was relatively less important for Callicebinae, Cebinae, and Pithecinae, affecting between 20.8% and 51.7% of their species. Similarly, while the effects of Development were third most common to primates generally, several Malagasy (Cheirogalaeinae, Daubentoniidae, Indriinae, Lemulirinae) and Neotropical taxa (Pitheciinae, Saimiriinae) were either not affected by Development, or it impacted only a relatively low number of species (Table I).

This pattern held when we examined the main Level 2 activities that were driving the two most common threats (i.e., Biological Resource Use and Agriculture; Table II) across taxonomic groups. For example, while hunting was one of the most common threats across most taxa, in several cases this threat affected only a relatively small proportion of species, including the Callitrichinae (*N* = 6 species, or 12.4%), Cheirogaleinae (*N* = 14 species, or 35%), Galagidae (*N* = 1 species, or 5.3%), and Saimiriinae (*N* = 1 species, or 20%). Similarly, Cebinae (*N* = 7 species, or 41.2%), Galagidae (*N* = 9 species, or 47.3%) and Pitheciinae (*N* = 4 species or 16.7%) were relatively less affected by Annual and Perennial Non-Timber Crop Production, the most prevalent Agricultural activity affecting primates globally (Table II).

### Modeling IUCN Red List Status

The retained model (AIC of 1166.714, compared to AIC = 1167.28 of the next best model and AIC = 1173.483 of the full model), showed that among the 12 Level 1 IUCN threat types, those that had the largest influence on determining a species’ conservation status were Human Intrusion & Disturbance, followed by Agriculture & Aquaculture and Climate Change & Severe Weather (Table III). In particular, the odds of being considered at a higher risk of extinction increased by 3.2 when a species was affected by Human Intrusion & Disturbance, by 2.6 when affected by Agriculture & Aquaculture, and by 2.3 when affected by Climate Change & Severe Weather (Table III). Region also had an effect: being from Madagascar increased the odds of being at higher risk of extinction by 4.9, while being from Asia increased the odds 3.3 times. Finally, increasing the number of countries in which a species was found decreased the odds of being at higher risk of extinction. None of the other variables had a significant effect predicting the conservation status of a species (Table III).

**Table III:** Retained model following a backward stepwise regression examining the variables affecting a species’ IUCN conservation status (excluding Data Deficient species)

Variable	Estimate (*b*)	Standard error	*t*-value	Odds ratio	*P*
Region					
*Asia*	*1.19*	*0.29*	*4.07*	*3.29*	*<0.001*
*Madagascar*	*1.60*	*0.33*	*4.86*	*4.93*	*<0.001*
The Americas	−0.21	0.30	−0.07	0.81	0.49
*Agriculture & Aquaculture*	*0.95*	*0.34*	*2.76*	*2.58*	*<0.01*
Biological Resource Use	0.61	0.33	1.86	1.84	0.06
*Human Intrusion & Disturbance*	*1.17*	*0.32*	*3.64*	*3.21*	*<0.001*
Habitat Modification	0.40	0.21	1.88	1.50	0.06
*Climate Change and Severe Weather*	*0.85*	*0.37*	*2.31*	*2.34*	*<0.05*
*Number of Countries*	*-0.18*	*0.04*	*−4.38*	*0.84*	*<0.001*

Variables included in the model were region in which the species is found (Africa, Asia, Madagascar, The Americas), whether the species was affected by any of the 12 IUCN Level 1 threat types (Yes/No), the number of countries in which the species was found, and the number of Level 1 threats described for the species. Italics indicate significant results.

The predictive probability of the ordinal logistic regression model based on the type and number of threats was poor, however, as the confusion matrix showed a predictive accuracy of only 41.2% (Table IV). The model did relatively well correctly classifying species that were Endangered (63.0%) and Least Concern (55.9%), but not for the other three conservation status categories. The model completely failed to correctly classify Near Threatened species based on the threats and regions of occurrence (Table IV).

**Table IV:** Confusion matrix showing predictive probability of the retained ordinal logistic regression model

Conservation status					
Predicted	Actual				
	Least Concern	Near Threatened	Vulnerable	Endangered	Critically Endangered
Least Concern	**33**	9	11	6	3
Near Threatened	0	**0**	0	0	0
Vulnerable	17	12	**24**	20	10
Endangered	9	5	37	**68**	39
Critically Endangered	0	0	8	14	**15**

Bolded values indicate number of correctly classified species.

#### Scientific Publications

Papers published between January 1, 2015 and July 1, 2020 revealed that the threats that affected the largest proportion of species were those related to habitat destruction, including Habitat Degradation (*N* = 168 species, or 58.5%); Hunting (*N* = 134 species, or 46.7%); Commercial & Small-Holder Agriculture (*N* = 102 species, or 35.5%); Urbanisation & Road Development (*N* = 98 species, or 34.1%); and Logging, Wood Harvesting, & Gathering of Terrestrial Plants (*N* = 88 species, or 30.7%) (Table V). In addition, threats that were relatively uncommon in the IUCN Red List were much more frequently mentioned in the recent literature (Table V), such as Climate Change & Severe Weather (*N* = 97 species, or 33.8%), Pet Trade (*N* = 73 species, or 25.4%), and Diseases (*N* = 62 species, or 21.6%).

**Table V:** Number of species within each region, primate group and overall affected by the main 12 threat categories identified while reviewing scientific articles published during the past 5 yr (January 1, 2015–July 1, 2020)

			Number of species	Urbanisation & Road Development	Commercial & Small-Holder Agriculture	Energy Production & Mining	Habitat Degradation	Logging, Wood Harvesting & Gathering of Terrestrial	Hunting	Pet trade	Civil Unrest	Genetics	Diseases	Climate Change & Severe Weather	Tourism
*Region*															
	Africa		69	25	28	19	39	26	44	14	1	2	16	20	19
	Asia		76	37	35	16	46	40	36	35	5	7	7	13	8
	Madagascar		70	12	18	2	30	7	18	6	2	1	11	56	4
	The Americas		72	24	21	10	53	15	36	18	0	7	28	8	2
		*Average*		24.5	25.5	11.8	42.0	22.0	33.5	18.3	2.0	4.3	15.5	24.3	8.3
		*SD*		8.8	6.6	6.5	8.5	12.4	9.5	10.6	1.9	2.8	7.9	18.8	6.6
*Taxon*															
	Cheirogaleidae		22	3	9	2	10	1	3	0	2	0	5	13	0
	Daubentoniidae		1	0	1	0	0	1	1	0	0	0	0	1	0
	Indriidae		13	1	3	0	4	1	4	1	0	0	2	12	0
	Lemuridae		21	8	3	0	13	3	8	5	0	1	4	19	4
	Lepilemuridae		13	0	2	0	3	1	2	0	0	0	0	11	0
	Galagidae		8	2	2	3	6	2	2	2	0	0	0	3	1
	Lorisinae		7	3	3	2	4	4	3	7	1	0	1	0	1
	Perodicticinae		2	0	1	1	0	1	0	1	0	0	0	1	0
	Tarsiidae		2	0	1	0	0	1	0	0	0	0	0	1	0
	Alouattinae		8	4	3	1	5	3	4	3	0	0	6	1	0
	Atelinae		8	2	5	2	7	3	6	1	0	0	4	1	0
	Aotinae		5	1	2	0	3	1	3	2	0	0	3	1	0
	Callitrichinae		16	7	2	2	12	1	8	8	0	4	6	0	0
	Cebinae		10	3	4	2	7	4	5	2	0	0	4	1	1
	Saimiriinae		5	0	0	0	2	0	1	1	0	0	2	1	0
	Callicebinae		14	3	3	2	11	2	5	0	0	3	3	2	0
	Pitheciinae		4	2	0	0	4	0	3	0	0	0	0	0	0
	Cercopithecini		25	11	15	7	13	12	17	5	0	0	4	4	9
	Papionini		29	17	11	7	17	10	19	8	0	1	8	11	7
	Colobinae		52	22	25	12	30	26	25	17	2	8	4	8	7
	Hylobatidae		15	4	5	2	12	8	9	7	2	0	2	3	1
	Ponginae		3	2	1	1	3	2	3	2	0	0	0	2	0
	Homininae		4	3	1	1	2	1	3	1	1	0	4	1	2
		*Average*		4.3	4.4	2.0	7.3	3.8	5.8	3.2	0.3	0.7	2.7	4.2	1.4
		*SD*		5.5	5.6	2.9	6.8	5.6	6.2	4.0	0.7	1.8	2.3	5.2	2.6
*Overall*															
	Total		287	98	102	47	168	88	134	73	8	17	62	97	33
	%			34.1	35.5	16.4	58.5	30.7	46.7	25.4	2.8	5.9	21.6	33.8	11.5

Primate groups included family, subfamily, and in the case of the subfamily Cercopithecinae, tribe.

We found similar differences at the regional level. For example, Climate Change & Severe Weather was relatively common in all four regions, particularly in Madagascar, where it was reported to affect 80% (*N* = 56) of the species studied (Table V). In contrast, in the IUCN Red List this threat was uncommon, and only in Africa was it relatively prevalent (Table IV). Likewise, the effect of Diseases was also relatively large in all regions in the recent literature compared to the IUCN Red List, with the The Americas (*N* = 28 species, or 38.9%) and Africa (*N* = 16 species, or 23.2%) having the largest proportion of species affected (Table V). Finally, according to recent publications, the effect of Energy Production & Mining projects was much less important in Madagascar than the IUCN Red List would suggest, with publications mentioning this threat only for 2 species (2.9%), compared to 31 species (29.0%) listed as threatened by Energy projects in the IUCN Red List (Table V).

Results by taxonomic group paralleled those found at the regional level. Compared to the IUCN Red List, primates were affected by a larger number of threats in the recent literature. In particular, 6 taxonomic groups were affected by 3–6 threats (Daubentoniidae, Lepilemuridae, Perodicticinae, Pitheciinae, Tarsiidae, and Saimiriinae), 9 by 7–9 threats (Alouattinae, Aotinae, Atelinae, Callicebinae, Callitrichinae, Cheirogaleidae, Galagidae, Indriidae, and Ponginae), and 8 by 10–12 threats (Cebinae, Cercopithecini, Colobinae, Lemuridae, Lorisinae, Homininae, Hylobatidae, and Papionini). Among them, Cebinae, Hylobatidae, Cheirogaleinae, and Lemuridae had between 3 and 5 more threats in the recent publications than indicated in the IUCN Red List. In contrast, Tarsiidae and Pitheciinae had 6 and 3 threat types fewer in the literature review compared to the IUCN Red List, respectively.

## Discussion

Our results confirmed that the majority of primate species are currently threatened and require urgent conservation attention. The IUCN Red List showed that the two main threats to primates worldwide are Biological Resource Use (including Hunting and Logging) and Agriculture. Although the IUCN Red List does not distinguish between small and large-scale commodity-driven agriculture, it is the latter that seems to pose the main threats to primate populations, given that it creates permanent forest clearings and networks of roads and railways and make intensive use of pesticides (Curtis *et al*., 2018; Estrada *et al*., 2017). Furthermore, it has been predicted that the growing demands for goods produced by commodity agriculture may lead to an agricultural expansion that will overlap with 68% of current primate distributions (Estrada *et al*., 2017).

Impact of these threats varied by region and primate group, with the Americas at greater risk due to Livestock Production compared to other regions. Our results also suggested that medium (5-10 kg) and larger-bodied (≥ 10kg) primates are facing a greater variety of threats compared to smaller-bodied primates. This is probably due to the many intrinsic factors that increase their risk of extinction, including attractiveness to hunters both for meat and for trophies, the need for more food resources, and longer life histories, such as later age at first birth, which can slow the pace of population growth (Isaac & Cowlishaw, 2004; McKinney, 1997; Purvis *et al*., 2000). Although our model to predict conservation status based on threats did not strongly predict Red List categories, it did clearly show that Malagasy and Asian primates are more likely to be listed as threatened compared to other regions. Our analysis of literature published in the past 5 yr highlighted that some threats, such as climate change and disease, were more prevalent in recent studies than in the Red List.

### Rethinking Primate Conservation Strategies

Our results demonstrated that the majority of primates are affected by more than 7 major threat types, with some, such as cercopithecines and colobines, facing 10 and 11 major threats, respectively. This highlights the need for varied and robust evidence-based conservation actions. The Conservation Evidence working group (www.conservationevidence.com) has evaluated the effectiveness of mitigation interventions focused on primates. Although there is a significant lack of such evidence in primatology (Junker *et al*., 2020a), there are some effective interventions addressing the main threats that many primate species face that conservation projects should prioritize. For example, interventions aimed at strengthening law enforcement, such as organizing well-equipped, regular antipoaching ranger patrols, conducting regular snare removal, implementing community-led no-hunting policies and patrols, and maintaining research sites with permanent human presence would be impactful for cercopithecines and colobines (Junker *et al*., 2020a, 2020b). Such projects should also include interventions aimed at protecting habitat and increasing connectivity, such as creating and protecting corridors and installing rope or pole canopy bridges above roads and railways, as well as interventions aimed at reducing the effect of human intrusion into primate habitat and promoting human–wildlife coexistence, such as deterrence of crop raiding using loud noises or other means (Junker *et al*., 2020a, 2020b).

#### ***Mitigation Recommendations Protected Areas***

Primate conservation needs a greater focus on the creation of new protected areas and on improving enforcement of current protected areas to safeguard preferred habitats, reduce habitat fragmentation and prevent illegal hunting and collection of primates. There is evidence that that improving enforcement of protected areas is more effective than most other initiatives (Junker *et al*., 2020a, 2020b). However, it is also important to recognise that many protected areas are essentially paper parks, lacking effective enforcement, and many primate populations are found outside of protected areas (Hoskins *et al*., 2020; Murai *et al*., 2013; N’Goran *et al*., 2012). Thus, this strategy alone will not be enough to safeguard the future of threatened primates.

#### Capacity Building and Codeveloping Strategies for Coexistence

As the majority of primates live outside protected areas, the conservation community and primate range states must put considerable effort into effectively supporting the sustainable use of natural resources for communities living alongside primate populations, as well as alternative livelihoods and codeveloping strategies to improve human–primate coexistence in shared landscapes. For example, a study in Batang Seangan region, in north Sumatra, found that using nets to cover the canopy of fruiting trees and driving away animals using loud noises, such as firecrackers and bamboo drums, was not only highly effective at reducing orangutan crop foraging, but it also decreased financial loss and changed farmer perception on animal conflict management, favoring mitigation rather than animal relocations (Campbell-Smith *et al*., 2012). Five months after the study, however, only 40% of farmers continued using mitigation strategies, and they all fall back on bamboo drums— which had little effect preventing crop foraging—over tree nets and firecrackers, citing that the former was less costly and simpler than the other two. This example highlights two key points: such measures can be effective to improve human–primate coexistence and reduce threats to primates; however, it can require long-term engagement with local farmers to maintain their impact and must be consistently monitored and evaluated to ensure its effectiveness and highlight areas for improvement. Thus, primatologists and conservationists working to reduce threats to primate populations must collaborate closely with local people to develop site-specific guidelines and facilitate their long-term adoption (Chan *et al*., 2007).

#### Lon-Term Funding for Underdeveloped Countries

While agriculture is a major threat affecting all primate regions, its impact in Madagascar is the largest. This may be a product of the underdevelopment of Madagascar and the level of poverty, where 75% of the population was estimated to live below the international poverty line (World Bank, 2019). In general, conservation granting bodies tend to preferentially fund initiatives in underdeveloped countries compared to middle income countries, and hundreds of millions of dollars have been invested in Madagascar since the early 1990s (Waeber *et al*., 2016). However, much of this funding has been awarded as small or medium-sized grants to fund short-term research projects that have had little impact on long-term primate species survival (Waeber *et al*., 2016). Unfortunately, stable long-term funding and the prioritization of self-sustaining projects, continues to be very limited and requires addressing within the global conservation funding community (Sodhi *et al*., 2011; Struhsaker *et al*., 2005)

### Predicting the Conservation Status of Data Deficient Species

Our model determined that the threat of Human Intrusion & Disturbance (such as civil unrest), Agriculture, and Climate Change & Severe Weather had the largest impact on determining a species conservation status. Region also had an impact in the model, as a species from Madagascar or Asia is almost 5 or 3 times more likely to be considered at higher risk of extinction compared to species from other regions, respectively. Perhaps appropriately, increasing the number of countries in which a species is found, and therefore the species’ distribution range, decreases the odds of having a higher conservation status.

As government agencies and conservation organisations use the IUCN Red List to guide their decision-making process, and funders tend to support work focused on threatened species over those whose status is unknown, primatologists should aim to fill the existing knowledge gap for these Data Deficient species. Our model would suggest, for instance, that current Data Deficient species affected by one or more of the threats with the largest impact determining a species’ conservation status, that are found in Madagascar or Asia, and with a distribution limited to one country should be assumed to be of higher risk of extinction and thus prioritized for immediate conservation action and research. For example, the Lariang tarsier (*Tarsius lariang*), native to Indonesia, is classified as Data Deficient, and affected by four different threats, including Agriculture (Shekelle *et al*., 2020). As all Tarsiidae for which we have a conservation status are classified as Near Threatened or above, our findings suggest that the Lariang tarsier should be considered Near Threatened at the very least, until further investigation into their populations can be conducted. Similarly, both the Heuglin’s patas monkey (*Erythrocebus poliophaeus*), found in Ethiopia, Sudan, and South Sudan (Gippoliti & Rylands, 2020), and the Vieira’s titi monkey (*Plecturocebus vieirai*), endemic to Brazil (Alonso *et al*., 2018), have a relatively small species distribution range and are affected by four different threat types, including Agriculture. Until further research can confirm the status of these two species, we would recommend the IUCN Red List consider them to be Vulnerable at the very least.

#### Limitations of the Current Model

Although these results seem promising for a predictive model to help with classifying poorly known species, the predictive power of the model was only 41.2%, which may mean more factors need to be considered or that other key factors may be more accurate predictors of conservation status (e.g., current area of occupancy, or key life history traits). Interestingly, a model to predict extinction risk in primates and carnivores by examining factors including range size and slow life histories explained nearly 50% of the variation (Purvis *et al.*, 2000). Thus, future studies should consider a hybrid model, combining threats, region, and intrinsic risk factors, to predict conservation status for unassessed primates.

The limitation of the current model may also be a product of the current process for assessing species on the Red List itself. The standard of data required to list a species as Critically Endangered, or to upgrade a species from Least Concern to one of the threatened categories, has become increasingly stringent. As previously mentioned, data required to make such justifications are often not collected in a standardized manner or are unknown across large parts of a species’ range. Even when experts may know, from working in a region or through anecdotal reports, that a species or its habitat is affected by particular threats, putting it at extremely high risk of extinction in the wild, such a threatened conservation status may not be approved. This is often due to a lack of solid evidence from research demonstrating a decrease in population size within three generations or significant reduction in the area of occupancy or extent of occurrence. Thus, a species that should be listed as Critically Endangered remains at a lower extinction risk level, limiting the conservation attention it may receive. This has been one of the criticisms of the Red List. Given the dearth of substantial quantitative data for many species and regions, some contributors have repeatedly advocated for more room for qualitative assessments (Mrosovsky, 2003; Tomasini, 2018), highlighting that we are running out of time to wait for ideal datasets and must make room for personal observations of nontraditional “experts” with valuable knowledge of species and the threats they face (Mrosovsky & Godfrey, 2008).

#### IUCN Red List Assessments and Emerging Threats

Another potential limitation of the IUCN Red List assessment system was highlighted when we compared threats identified in the Red List with those in recent literature. Climate change was a key factor in our model predicting a higher conservation status among primates on the Red List. However, in our analysis of the threats facing primates using IUCN data, climate change was listed for fewer than half of groups used in the present study, impacting an average of 11.3% of species within each of the groups. By contrast, it was mentioned in the past 5 yr of published literature for almost all of the primate groups, affecting approximately one third of species within each group. In fact, >90% of Lemuridae were reported to be impacted by climate change in the recent literature, while only 19% had this noted as a threat on the Red List. This is supported by a recent study that found the potential for considerable loss of or compromised habitat for nonhuman primates on a global scale, due to the emergence of climate conditions that are outside of the scope of historical experience for many species (Stewart *et al.*, 2020).

We found a similar pattern with disease, impacting an average of one quarter of species within almost every primate group in the literature but only an average of 8.5% of species on the Red List. More specifically, only 7% of Alouattinae are listed as threatened by disease on the Red List, while 75% of species were listed in the literature. For example, between 2007 and 2009 Argentina suffered a serious yellow fever epidemic that decimated brown howler monkey (*Alouatta guariba clamitans*) populations (Moreno *et al.*, 2015). The authors also state that the genus *Alouatta* is the most susceptible primate in the Americas to this disease, showing acute forms with high mortality, supporting our finding that it is a prevalent threat in the recent literature. Given the current Covid-19 pandemic, the risk of infectious disease among primates will surely become more and more prevalent.

The reasons for differences between the recent literature and the Red List are not entirely clear. One possibility may be that manuscripts often list potential threats, such as generic threats that have been described for a particular region or habitat, to their study species without citations for studies that have investigated those threats explicitly, but rather based on anecdotal evidence. In fact, most of the papers analyzed for this study were not conservation focused and threats were often listed in the abstract or methods under the description of the study species. This would mean that some of the 12 threat categories we identified from the literature may not be as prevalent as our study would suggest. Alternatively, the disparity between our literature review and the IUCN Red List may indicate that threats such as climate change and disease are emerging threats to primates and therefore we lack solid evidence to definitely list them as major threats on the Red List, which is data driven and based on objective criteria for estimating extinction risk (Rodrigues *et al*., 2006). However, the repeated mention of these threats in current literature may indicate that those researchers currently working with these taxa are beginning to notice significant impacts on the ground.

One additional, and perhaps related, explanation is likely the continuing disconnect between research scientists and applied conservationists. For decades it has been highlighted that more collaboration is required to bring the benefits of behavioral ecology research to the conservation arena, as well as to have the applied conservation practitioners in the discussions about the direction and design of current research in order to fill in the knowledge gaps and deliver research with impact (Angeloni *et al*., 2008; Berger-Tal *et al*., 2011; Caro, 1999, 2007; Cooke *et al*., 2014; Saterson *et al*., 2004). Each of these papers analyzes whether these two sides are working collaboratively for the benefit of conservation, and to date, their conclusion continues to be that they are not. Many Red List contributors are applied conservation professionals, rather than early career researchers who tend to lead on current publications. The disconnect in threats facing some of these primates may be addressed by greater inclusion of up-and-coming researchers into the Red Listing process, as well researcher engagement with applied conservation professionals prior to publication, to ensure that listed threats are in fact verified by those working on the frontlines of conservation.

## Conclusion

In conclusion, primates remain one of the most threatened mammalian groups on the planet. Although all regions face threats, species in Madagascar and Asia are at highest risk of extinction, requiring urgent attention, as they are under siege from hunting, the pet trade, logging, and non-timber crop production. The global primate conservation community must also be vigilant to emerging threats as well, such as climate change and infectious disease. We must work collectively, both researchers and applied conservationists, to develop evidence-based conservation initiatives that are both robust and nuanced, to be able to meet the varied challenges facing primates around the world and reverse the trend of increasing rates of threatened species on the IUCN Red List. 

## Supplementary Information

ESM 1 (PDF 175 kb)

ESM 2 (PDF 203 kb)

ESM 3 (PDF 163 kb)
