# Supplementary material for: The Current Status of the World’s Primates: Mapping Threats to Understand Priorities for Primate Conservation
Source: Int J Primatol. 2021 Oct 31;43(1):15–39. doi: 10.1007/s10764-021-00242-2 (PMC8557711; doi:10.1007/s10764-021-00242-2)
Supplement: Supplementary file 1 — (PDF 175 kb) [file 10764_2021_242_MOESM1_ESM.pdf]

Table SI Keywords and the 12 predefined, higher-level primate threat categories identified after the preliminary literature review. These keywords and threat categories were used to perform the literature review to examine if there were new and up-and-coming threats that had become more prevalent after the last IUCN Red List assessment.

**Urbanisation & Road Development**	**Commercial Agriculture**	**Small Holder Agriculture**	**Energy Production & Mining**	**Logging, Wood Harvesting & Gathering Terrestrial Plants**	**Commercial Hunting**	**Subsistence Hunting**	**Pet Trade**	**Civil Unrest**	**Genes**	**Diseases**	**Climate Change & Severe Weather**
Road	Industrial agriculture	Illegal crop cultivation	Hydrocarbon	Logging	Commercial bushmeat	Subsistence hunting	Pet trade	Civil unrest	Inbreeding	Disease	Climate change
Rail	Clearing for agriculture	Slash and burn	Oil	Wood extraction	Commercial hunting		Primate trade	Political instability	Hybridisation	Parasite	Climatic variability
Human population	Large-scale agriculture	Pastoral	Gas	Wood harvesting	Illegal wildlife trade		Illegal trade	Corruption		Parasitic agent	Climatic variation
Illegal settlement	Plantation	Terracing	Mining	Timber exploitation			Traditional medicine	Landmine		Pathogen	Changing climatic condition
Expansion of urban area	Ranching	Garden	Dam	Timber extraction			Wildlife laundering	Bombing		Virus	Micro-climate change
Infrastructure development	Agro-industry	Horticulture	Hydroelectric	Timber cutting				Poor governance			Extreme climatic event
Encroachment	Shifting agriculture		Extraction	Tree removal				War			Global climatic change
Urbanization	Soy bean		Pylon collision	Fuelwood gathering							Global warming
	Palm oil		Electrocution	Hardwood							Localised natural disaster
	Sugar cane		Power-line	Fire wood							Natural disaster
	Rice		Charcoal production	Destruction of forest undergrowth							Cyclone
	Cotton			Intensive fuelwood harvesting							Excess precipitation
	Industrial farming										Modified rainfall pattern

	Large-scale farming										Changing rainfall pattern
											Extreme rain
											Declining water balance
											Flood
											Hurricane
											Increased temperature
											Elevated temperature
											Drought
