# Supplementary material for: The Current Status of the World’s Primates: Mapping Threats to Understand Priorities for Primate Conservation
Source: Int J Primatol. 2021 Oct 31;43(1):15–39. doi: 10.1007/s10764-021-00242-2 (PMC8557711; doi:10.1007/s10764-021-00242-2)
Supplement: Supplementary file 2 — (PDF 203 kb) [file 10764_2021_242_MOESM2_ESM.pdf]

Table SII Search terms, keywords, and speech marks used to perform the literature review. For each threat category, we used approximately 10% of all the Google Scholars results, selecting only research articles that discussed primate conservation threats (*N* = 899 articles). Once duplicates were eliminated, we ended up with 398 articles in total.

**Category**	**Search terms and key words**	**No. of Google Scholar results**	**No. of articles collected (10%)**
Urbanisation & Road Development	Road OR Rail OR "Human population" OR “Illegal settlement” OR “Expansion of urban area” OR “Infrastructure development” OR Encroachment OR Urbanization OR Urbanisation and "Primate conservation" and threat	1190	119
Commercial Agriculture	“Industrial agriculture” OR “Clearing for agriculture” OR “Large-scale agriculture” OR Plantation OR Ranching OR Agro-industry OR “Shifting agriculture” OR “Soy bean” OR “Palm oil” and "Primate conservation" and threat	575	58
	“Sugar cane” OR Rice OR Cotton OR “Industrial farming” OR “Large-scale farming” and "Primate conservation" and threat	558	56
Small Holder Agriculture	“Illegal crop cultivation” OR “Slash and burn” OR Pastoral OR “Terracing” OR Garden OR Horticulture and "Primate conservation" and threat	515	52
Energy Production & Mining	Hydrocarbon OR Oil OR Gas OR Mining OR Dam OR Hydroelectric OR Extraction OR “Pylon collision” OR Electrocution OR Power-line OR “Charcoal production” and "Primate conservation" and threat	1240	124
Logging, Wood Harvesting & Gathering Terrestrial Plants	Logging OR “Wood extraction” OR “Wood harvesting” OR “Timber exploitation” OR “Timber extraction” OR “Timber cutting” OR “Tree removal” OR “Fuelwood gathering” OR “Hardwood” and "Primate conservation" and threat	917	92
	“Fire wood” OR “Destruction of forest undergrowth” OR “Intensive fuelwood harvesting” and "Primate conservation" and threat	27	3
Commercial Hunting	“Commercial bushmeat” OR “Commercial hunting” OR “Illegal wildlife trade” and "Primate conservation" and threat	190	19
Subsistence Hunting	"Subsistence hunting" and "Primate conservation" and threat	133	13
Pet Trade	“Pet trade” OR “Primate trade” OR “Illegal trade” OR “Traditional medicine” OR “Wildlife laundering” and "Primate conservation" and threat	464	46
Civil Unrest	“Civil unrest” OR “Political instability” OR Corruption OR Landmine OR Bombing OR “Poor governance” OR War and "Primate conservation" and threat	637	64
Genes	Inbreeding OR Hybridisation and "Primate conservation" and threat	251	25
Diseases	Disease OR Parasite OR “Parasitic agent” OR Pathogen OR Virus and "Primate conservation" and threat	1010	101
Climate Change & Severe Weather	“Climate change” OR “Climatic variability” OR “Climatic variation” OR “Changing climatic condition” OR “Micro-climate change” OR “Extreme climatic event” OR “Global climatic change” and "Primate conservation" and threat	711	71
	“Global warming” OR “Localised natural disaster” OR “Natural disaster” OR Cyclone OR “Excess precipitation” OR “Modified rainfall pattern” OR “Changing rainfall pattern” OR “Extreme rain” and "Primate conservation" and threat	186	19
	“Declining water balance” OR Flood OR Hurricane OR “Increased temperature” OR “Elevated temperature” OR Drought and "Primate conservation" and threat	373	37
