# Supplementary material for: The Current Status of the World’s Primates: Mapping Threats to Understand Priorities for Primate Conservation
Source: Int J Primatol. 2021 Oct 31;43(1):15–39. doi: 10.1007/s10764-021-00242-2 (PMC8557711; doi:10.1007/s10764-021-00242-2)
Supplement: Supplementary file 3 — (PDF 163 kb) [file 10764_2021_242_MOESM3_ESM.pdf]

Table SIII Twelve larger threat categories and their corresponding specific threat types identified during the literature review of 398 research articles (peer-reviewed papers, PhD and master's theses) that focused on conservation threats to primates published between January 1, 2015 and July 1, 2020.

**Urbanisation & Road Development**	**Agriculture**	**Habitat degradation**	**Energy Production & Mining**	**Logging, Wood Harvesting & Gathering Terrestrial Plants**	**Hunting**	**Tourism**	**Pet Trade**	**Civil Unrest**	**Genes**	**Diseases**	**Climate Change & Severe Weather**
Road	Industrial agriculture	Habitat reduction	Hydrocarbon	Logging	Commercial bushmeat	People's perceptions	Pet trade	Civil unrest	Inbreeding	Disease	Climate change
Rail	Clearing for agriculture	Habitat disturbance	Oil	Wood extraction	Commercial hunting	Ecotourism	Primate trade	Political instability	Hybridisation	Parasite	Climatic variability
Human population	Large-scale agriculture	Habitat modification	Gas	Wood harvesting	Illegal wildlife trade	Tourism-related disturbance.	Illegal trade	Corruption	Low genetic diversity	Parasitic agent	Climatic variation
Illegal settlement	Plantation	Habitat fragmentation	Mining	Timber exploitation	Subsistence hunting	Photography	Traditional medicine	Landmine		Pathogen	Changing climatic condition
Expansion of urban area	Ranching	Habitat loss	Dam	Timber extraction	Hunting	Tourism	Wildlife laundering	Bombing		Virus	Micro-climate change
Infrastructure development	Agro-industry	Habitat degradation	Hydroelectric	Timber cutting	Poaching		Illegal trade	Poor governance			Extreme climatic event
Encroachment	Shifting agriculture	Habitat destruction	Extraction	Tree removal	Shootings		Illegal animal trade	War			Global climatic change
Urbanization	Soy bean	Changes to habitat	Pylon collision	Fuelwood gathering	Hunting for meat consumption		Wildlife trade	Poverty			Global warming
Motor vehicle collisions	Palm oil	Large-scale tropical forest disruption	Electrocution	Hardwood	Hunting pressure						Localised natural disaster

Domestic cats and dogs	Sugar cane	Increasing land-use pressure and increasing demands for forest resources, resulting in deforestation, degradation and further fragmentation	Power-line	Fire wood	Hunting						Natural disaster
Monkey-dog conflict	Rice	Land use changes	Charcoal production	Destruction of forest undergrowth	Capture						Cyclone
Dog predation	Cotton	Human-modified landscapes		Intensive fuelwood harvesting	Illegal poaching						Excess precipitation
Attack by shepherd dogs	Industrial farming	Human-induced habitat disturbance		Illegal bamboo harvesting	Illegal traps						Modified rainfall pattern
Dogs	Large-scale farming	Unregulated land use		Ethnobotanical exploitation of resources	Illegal hunting						Changing rainfall pattern
Feral cats	Illegal crop cultivation	Deforestation causes flooding which gives lead poisoning			Meat						Extreme rain
	Slash and burn	Deforestation			Illegal capture						Declining water balance
	Pastoral	Over-exploitation of forest			Hunting for meat						Flood
	Terracing	Forest clearance									Hurricane
	Garden	Forest disturbance									Increased temperature
	Horticulture	Clear-cutting									Elevated temperature
	Farming	Forest fragmentation									Drought
